# Correspondence

**Published:** 1885-10

**Authors:** 


CORRESPONDENCE.—The following letter was received from a prominent dental practitioner of Georgia for publication:

AUGUSTA, Sept. 30th, 1885.

EDITOR "AM. JOURNAL OF DENTAL SCIENCE":

DEAR SIR: According to my knowledge of the proceedings of the organizations known as the "National Board of Dental Examiners," and the "National Association of Dental Faculties," it was determined that no dental school would be regarded as reputable that did not after June, i885, require TWO FULL SESSIONS OF FIVE MONTHS EACH IN SEPARATE YEARS FOR GRADUATION. The only exceptions made being those who after graduation in medicine had passed one year in the study and practice of clinical dentistry, and also those who had attended a previous session at a reputable dental school. I believe that the American Dental Association also adopted the same rule. Am I not correct? I therefore ask how it is that the dental school of Vanderbilt University is permitted to offer graduation at the close of but one session, to a student of this city who has passed one session only, and that very irregularly, at the Georgia Medical College? I also ask how the same school can offer similar inducements to another student from Edgefield, South Carolina, as I understand it has done, and yet be declared reputable? Was it for for the purpose of permitting such violations of the rules adopted by the different organizations referred to, that the "National Association of Dental Faculties" allowed the dental school of Vanderbilt University to abstain from becoming a member of that Association for the present year, and accorded to its Dean the privileges of the floor at its late meeting in Chicago? I cannot see why some schools should be compelled to conform to a rule that others may violate with impunity, and I think that the State Boards of Dental Examiners of both my own state and South Carolina should investigate the matter and act accordingly.

Respectfully, &c.,

"JUSTICE."

We can only reply to the above letter by stating that several students who as we had learned from their preceptors, intended to matriculate in the Dental Department of the University of Maryland, on discovering that they would be required to attend two sessions in the institution, had, we are informed, been induced to go to Vanderbilt by the promise of graduation on one session's attendance.

EDITOR OF "AM. JOURNAL OF DENTAL SCIENCE."

